# Meta-Analysis of EGFR Tyrosine Kinase Inhibitors Compared with Chemotherapy as Second-Line Treatment in Pretreated Advanced Non-Small Cell Lung Cancer

**DOI:** 10.1371/journal.pone.0102777

**Published:** 2014-07-16

**Authors:** Ning Li, Lu Yang, Wei Ou, Liang Zhang, Song-liang Zhang, Si-yu Wang

**Affiliations:** 1 Department of Thoracic Surgery, Sun Yat-sen University Cancer Center, Guangzhou, China; 2 Department of Breast Oncology, Sun Yat-sen University Cancer Center, Guangzhou, China; 3 Department of Thoracic Surgery, Tianjin First Center Hospital, Tianjin, China; Cardiff University, United Kingdom

## Abstract

**Background:**

Since efficacy and safety of epidermal growth factor receptor tyrosine kinase inhibitors (EGFR-TKIs) versus chemotherapy in the treatment of patients with pretreated advanced non-small cell lung cancer (NSCLC) remain controversial, we performed a meta-analysis to compare them.

**Methods:**

An internet search of several databases was performed, including PubMed, Embase, and the Cochrane database. Randomized trials that compared an EGFR-TKI with chemotherapy in the second-line setting were included. The outcomes were progression-free survival (PFS), overall survival (OS), objective response rate (ORR), and grade 3–4 toxicities. The PFS, OS for the EGFR mutation-positive (EGFR M^+^) and EGFR mutation-negative (EGFR M^−^) subgroups were pooled. The pooled hazard ratios (HRs) and odds ratios (ORs) with their corresponding confidence intervals (CIs) were calculated on the STATA software.

**Results:**

Our meta-analysis combined 3,825 patients from 10 randomized trials. Overall, EGFR-TKIs and second-line chemotherapy have equivalent efficacy in terms of PFS (HR, 1.03; 95%CI, 0.87–1.21; P = 0.73; I^2^ = 78.7%, P_heterogeneity_<0.001), OS (HR, 1.00; 95%CI, 0.92–1.08; P = 0.90; I^2^ = 0.0%, P_heterogeneity_ = 0.88), and ORR (OR, 1.34; 95%CI, 0.86–2.08; P = 0.20; I^2^ = 73.1%, P_heterogeneity_<0.001). However, subgroup analysis based on EGFR mutation status showed that second-line chemotherapy significantly improved PFS (HR, 1.35; 95%CI, 1.09–1.66; P = 0.01; I^2^ = 55.7%, P_heterogeneity_ = 0.046) for EGFR M^−^ patients, whereas OS was equal (HR, 0.96; 95%CI, 0.77–1.19; P = 0.69; I^2^ = 0.0%, P_heterogeneity_ = 0.43); EGFR-TKIs significantly improved PFS (HR, 0.28; 95%CI, 0.15–0.53; P<0.001; I^2^ = 4.1%, P_heterogeneity_ = 0.35) for EGFR M^+^ patients, whereas OS was equal (HR, 0.86; 95%CI, 0.44–1.68; P = 0.65; I^2^ = 0.0%, P_heterogeneity_ = 0.77). Compared with chemotherapy, EGFR-TKIs led to more grade 3–4 rash, but less fatigue/asthenia disorder, leukopenia and thrombocytopenia.

**Conclusions:**

Our analysis suggests that chemotherapy in the second-line setting can prolong PFS in EGFR M^−^ patients, whereas it has no impact on OS. EGFR-TKIs seem superior over chemotherapy as second-line therapy for EGFR M^+^ patients. Our findings support obtaining information on EGFR mutational status before initiation of second-line treatment.

## Introduction

Lung cancer remains the leading cause of cancer death in the world and approximately accounts for 13% of total cases and 18% of total deaths globally [1]. Although patients received standard first-line chemotherapy, most of them progressed ultimately. Docetaxel is considered as standard second-line treatment of advanced non-small-cell lung cancer (NSCLC) [2], [3]. Pemetrexed was approved for second-line treatment of advanced NSCLC after findings of a phase III trial by Hanna et al. showed equivalent outcomes. Pemetrexed was associated with few adverse events compared with docetaxel and comparable efficacy [4].

Epidermal growth factor receptor tyrosine kinase inhibitors (EGFR-TKIs, including Erlotinib and Gefitinib) have been approved as second-line therapy [5], [6], [7]. The BR.21 trial reported prolonged survival with erlotinib compared with placebo (median survival, 7.9 versus 3.7 months) in patients with advanced NSCLC after failure of previous chemotherapy [5].

However, the debate on the selection of EGFR-TKIs or chemotherapy in the second-line setting has heated up, even though several meta-analyses have been performed to address this issue. The editorial in 2012 gave an illustration of this debate [8]. Although the meta-analysis by Qi et al. demonstrated both EGFR-TKIs and chemotherapy had comparable efficacy in the second-line setting, the potential effect of EGFR mutation status on survival was not analysed [9]. The subsequent comprehensive meta-analysis by Lee et al. showed that an EGFR mutation is a predictive marker of PFS with EGFR-TKIs in all settings, but it included only 5 studies comparing EGFR-TKIs with chemotherapy in the second-line setting [10]. Recently, several trials showed that chemotherapy had superiority in progression-free survival (PFS) over EGFR-TKIs for EGFR mutation-negative (EGFR M^−^) patients [11], [12], [13]. A meta-analysis which included 3 trials in the 2013 ASCO annual meeting demonstrated chemotherapy can improve PFS compared with EGFR-TKIs for EGFR M^−^ patients [14]. To further investigate the optimal treatment and the role of EGFR mutation status in second-line setting, we performed this meta-analysis to compare the efficacy and safety of EGFR-TKIs versus chemotherapy as second-line treatment for pretreated advanced NSCLC.

## Methods

### Search Strategy

An internet search of PubMed, the Embase database, the Cochrane Central Register of Controlled Trials database (CENTRAL), the American Society of Clinical Oncology (ASCO), the European Society for Medical Oncology (ESMO) and the World Conference of Lung Cancer (WCLC) was performed in July 2013, via the various combinations of the following terms: “lung cancer”, “gefitinib”, “erlotinib”, “EGFR-TKI”, “second-line”, “randomized”. The language was limited to English. The relevant review articles and meta-analyses concerning the second-line treatment for patients with lung cancer were examined for inclusive trials and were listed.

### Selection Criteria

The relevant clinical trials were included if they met the following criteria: (1) they compared an EGFR-TKI with standard second-line chemotherapy (docetaxel or pemetrexed); (2) they were prospective randomized controlled trials (RCTs); (3) enrolled patients were previously treated with platinum compounds; (4) they reported sufficient data for extraction or sufficient data to calculate the effect measure. Two reviewers (L.N. and Y.L.) independently screened each reference to assess their eligibility for inclusion with disagreements settled by the third reviewer (W.SY.) until a consensus was reached.

### Data Extraction

Information from studies was extracted independently by 2 researchers (L.N. and Y.L.) and the following data were collected: publication details (such as the first author’s last name, year of publication, country in which the study was performed), trial information (such as study design, inclusion criteria, the number of the patients, chemotherapy regimens, type of end point used), patient characteristics (such as age, gender, stage, EGFR mutation status), outcome measures (such as HRs for PFS and OS and their 95%CIs, log-rank test P values, grade 3–4 adverse events). PFS and overall survival (OS) were defined as starting from randomization. The quality of the study was assessed on the Jadad score [15] to assess the trials according to randomization (0–2), appropriate blinding method (0–2), withdrawals and dropouts (0–1). The information extracted by the two researchers achieved excellent consistence.

### Statistical Analysis

The analysis was undertaken on an intention-to-treat basis. The results were presented as hazard ratios (HRs) and odds ratios (ORs) with their corresponding confidence intervals (CIs). For time-to-event data, the HRs and their 95%CIs were estimated by the methods proposed by Tierney et al. in the absence of published HRs or their CIs [16]. The summary HRs and their 95%CIs were estimated by a general variance-based method. The drug-related adverse events (AEs) were analyzed as grades 3 or above toxicity according to the National Cancer Institute common toxicity criteria (NCI-CTC) version 3. ORs were computed for dichotomous variables by the methods reported by Mantel and Haenszel [17]. Preplanned subgroup analyses to explore potential effect on PFS, OS based on EGFR mutation status were scheduled. Heterogeneity of the treatment effect between studies was estimated on the Q statistic and the heterogeneity I^2^ statistic [18]. If heterogeneity was considered statistically significant, random effects models were used and otherwise fixed effects models were used. Egger’s test and Begg’s funnel plots were used to check for potential publication bias [19], [20]. All the reported P values are 2-sided. STATA (version 12.0) was used for all analyses.

## Results

### Characteristics of the Included Studies

**Figure 1:**
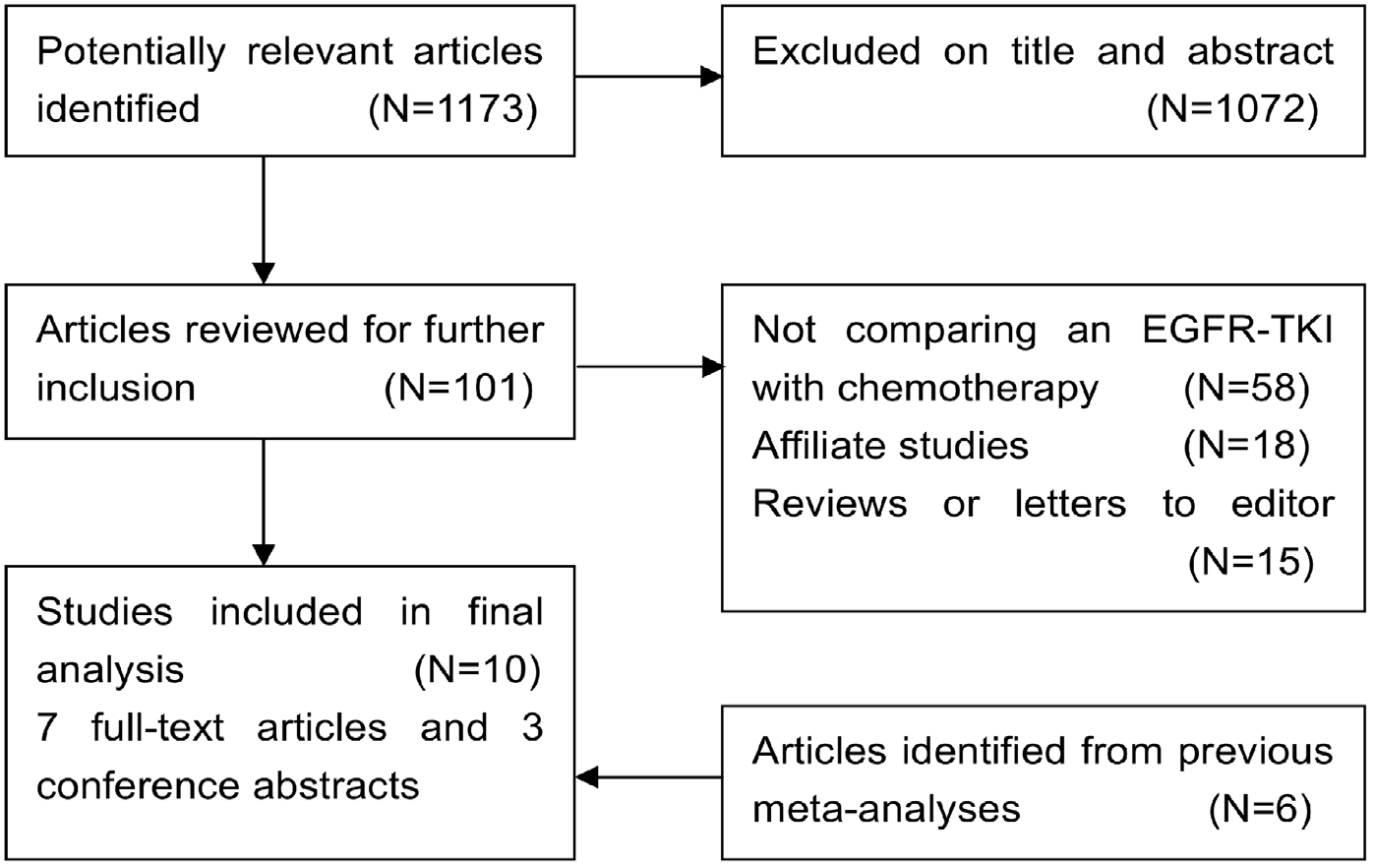
Flow diagram of trial identification process.

A total of 10 publications were included in the analysis, of which 6 trials [7], [21], [22], [23], [24], [25] were identified from previous meta-analyses, 4 trials [11], [12], [13], [26] were identified from internet searching. The flow diagram of our study is shown in Figure 1. Seven trials were reported in full text [7], , and the other 3 in conference abstracts [11], [12], [13]. The total number of randomized patients in these trials was 3825, with 1905 in the EGFR-TKI arm and 1920 in the chemotherapy arm. The total number of randomized patients of each trial ranged from 135 to 1466. None of the 10 included trials were placebo-controlled double-blinded trials and therefore none of them scored Jadad score 4 or above. Eight [7], [11], of the 10 trials were phase III RCTs, and the other 2 trials [13], [21] were phase II trials. Four trials [21], [22], [23], [24] compared gefitinib and docetaxel, 2 [11], [12] compared erlotinib and docetaxel, 2 [13], [25] compared gefitinib and pemetrexed, 1 [26] compared erlotinib and pemetrexed, and 1 [7] compared erlotinib and docetaxel/pemetrexed. The baseline characteristics of these studies are listed in Table 1. This meta-analysis followed the guidelines of the Preferred Reporting Items for Systematic Review and Meta-analyses (PRISMA) statement. The PRISMA Flow Diagram and Checklist are shown in Figure S1 and Checklist S1.

**Table 1:** Information of trials included in this meta-analysis.

Study/Year	Phase	Country	Therapy	N	Male (%)	Ever smoker (%)	IIIB (%)	IV (%)	EGFR M+ (%)	PFS (mo)	OS (mo)	RR (%)	Jadad score
SIGN, 2006	II	International	Gefitinib	68	30.9	67.6	39.7	60.3	NR	3.0	7.5	13.2	3
			Doc	73	30.1	67.1	43.8	56.2	NR	3.4	7.1	13.7	
INTEREST, 2008	III	International	Gefitinib	733	36.4	79.8	25.0	52.9	15.6	2.2	7.6	9.1	3
			Doc	733	33.4	79.6	28.8	52.3	14.1	2.7	8.0	7.6	
V-15-32, 2008	III	Japan	Gefitinib	245	38.4	71.0	19.2	64.9	NR	2.0	11.5	22.5	3
			Doc	244	38.1	64.3	20.5	61.5	NR	2.0	14.0	12.8	
ISTANA, 2010	III	Korea	Gefitinib	82	32.9	63.4	13.4	86.6	NR	3.3	14.1	28.1	3
			Doc	79	43.0	54.4	17.7	82.3	NR	3.4	12.2	7.6	
TITAN, 2012	III	International	Erlotinib	203	20.6	85.2	20.2	79.8	3.4	1.5	5.3	7.9	3
			Doc/Pem	221	27.6	80.1	23.1	76.9	1.8	2.0	5.5	6.3	
KCSG-LU08-01, 2012	III	Korea	Gefitinib	68	85.3	0	8.8	91.2	23.5	9.0	22.2	58.8	3
			Pem	67	85.1	0	9.0	91.0	25.4	3.0	18.9	22.4	
TAILOR, 2012	III	Italy	Erlotinib	109	29.4	81.7	NR	NR	0	2.4	NR	2.2	3
			Doc	110	33.6	71.8	NR	NR	0	3.4	NR	13.9	
HORG, 2013	III	Greece	Erlotinib	166	18.7	74.7	7.2	92.8	8.1	3.6	8.2	9.0	3
			Pem	166	16.9	77.1	11.4	88.6	9.8	2.9	10.1	11.4	
DELTA, 2013	III	Japan	Erlotinib	150	NR	NR	NR	NR	27.3	2.0	14.8	17.0	3
			Doc	151	NR	NR	NR	NR	40.4	3.2	12.2	17.9	
CTONG0806, 2013	II	China	Gefitinib	81	33.3	59.3	4.9	95.1	0	1.6	NR	13.6	3
			Pem	76	38.2	42.1	13.2	86.8	0	4.8	NR	13.2	

Abbreviations: N, number of patients; IIIB, stage IIIB; IV, stage IV; EGFR M^+^, epidermal growth factor receptor mutation-positive; PFS, progression-free survival; mo, month; OS, overall survival; RR, response rate; Doc, docetaxel; Pem, pemetrexed; NR, no report.

### Efficacy Analysis Results

**Figure 2:**
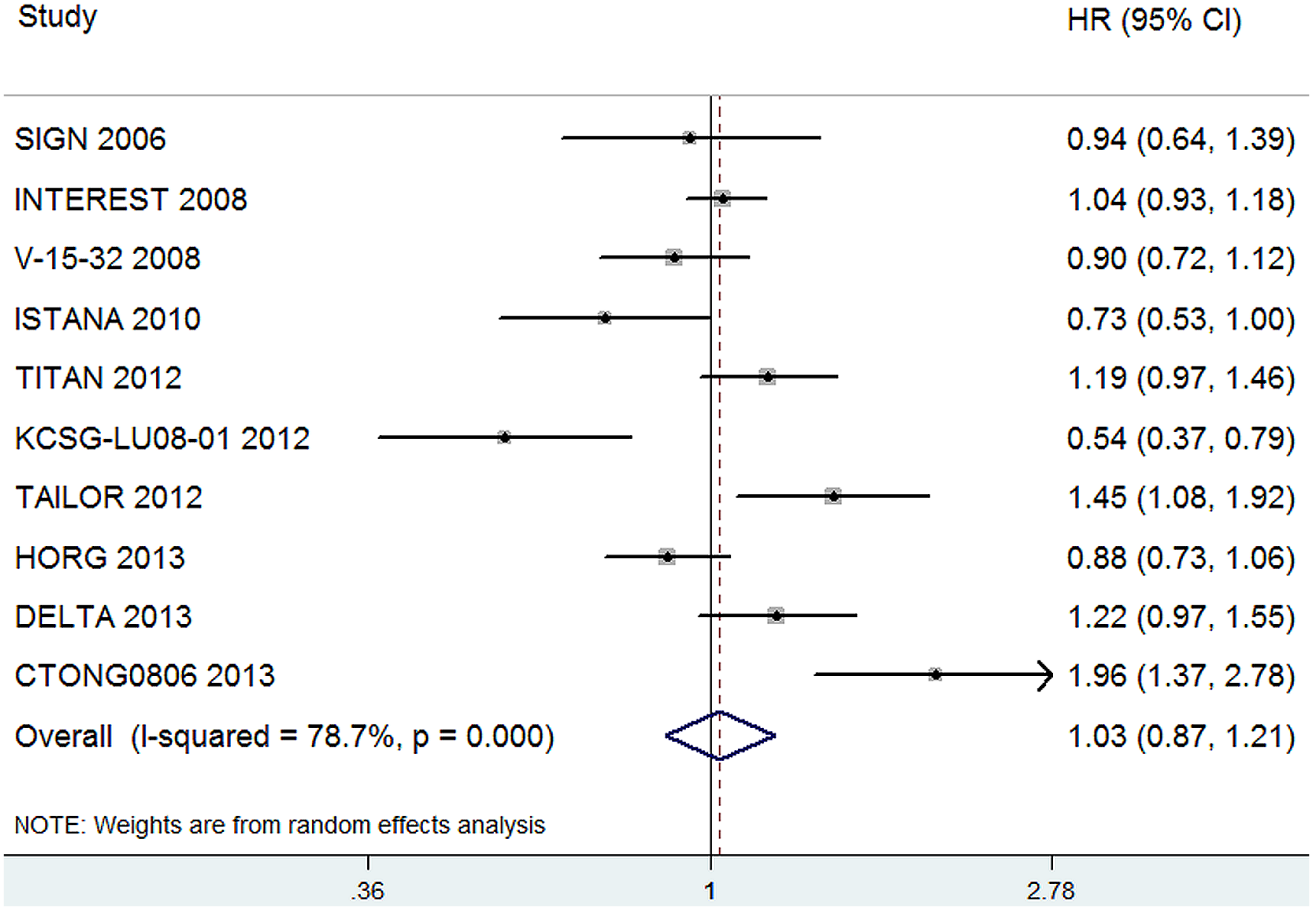
Comparison of PFS between EGFR-TKIs and chemotherapy.

All 10 trials reported PFS data. Overall, the pooled hazard ratio for PFS showed that there was no significant difference between an EGFR-TKI and second-line chemotherapy (HR, 1.03; 95%CI, 0.87–1.21; P = 0.73, Figure 2). Random effect model was used since heterogeneity across the trials was significant (I^2^ = 78.7%, P<0.001).

**Figure 3:**
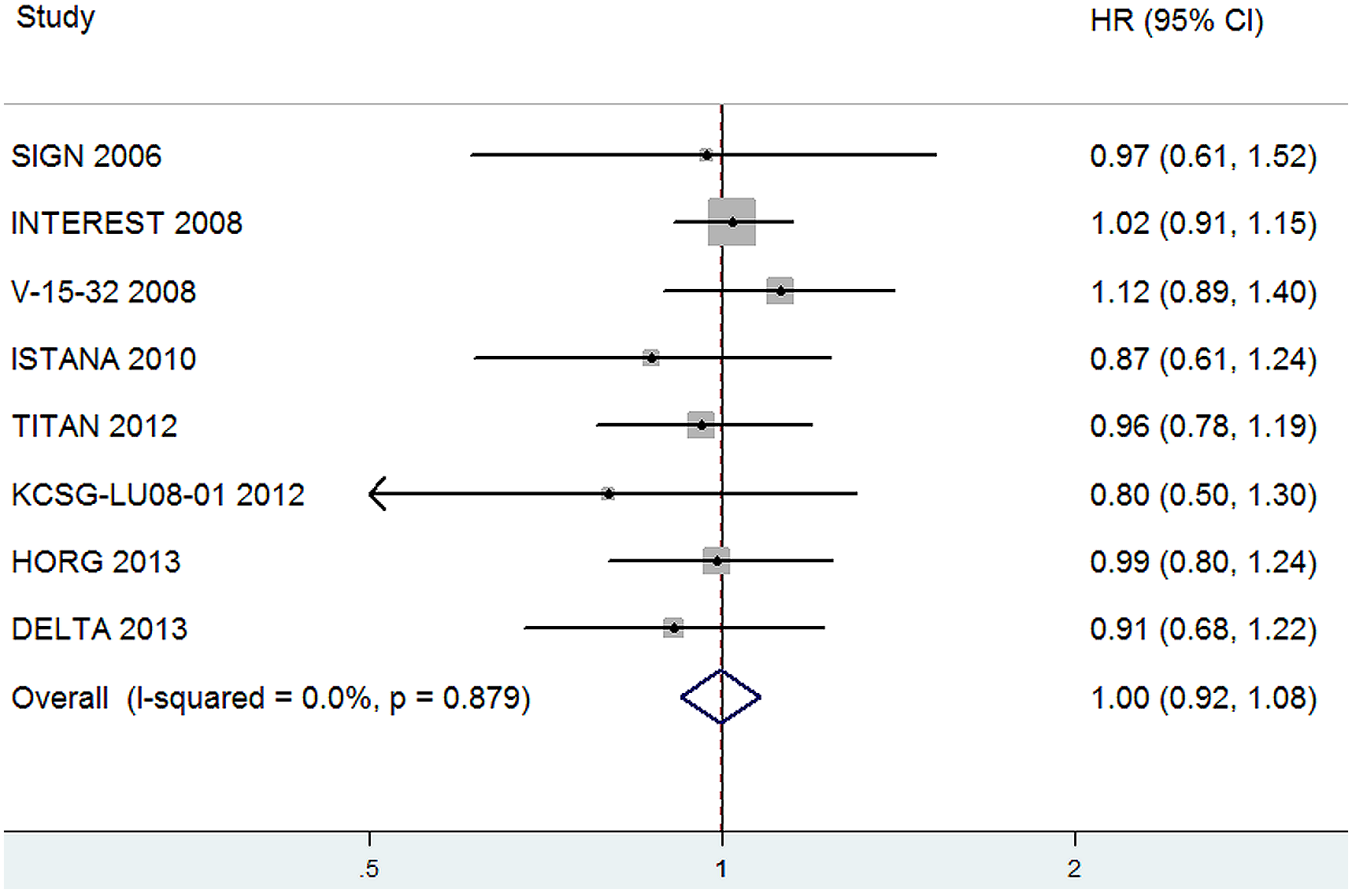
Comparison of OS between EGFR-TKIs and chemotherapy.

Data for OS were available from 8 trials [7], [12], [21], [22], [23], [24], [25], [26], and the pooled HR for OS showed that there was no significant difference between an EGFR-TKI and second-line chemotherapy (HR, 1.00; 95%CI, 0.92–1.08; P = 0.90, Figure 3). Fixed effect model was used since heterogeneity across the trials was not significant (I^2^ = 0.0%, P = 0.88).

**Figure 4:**
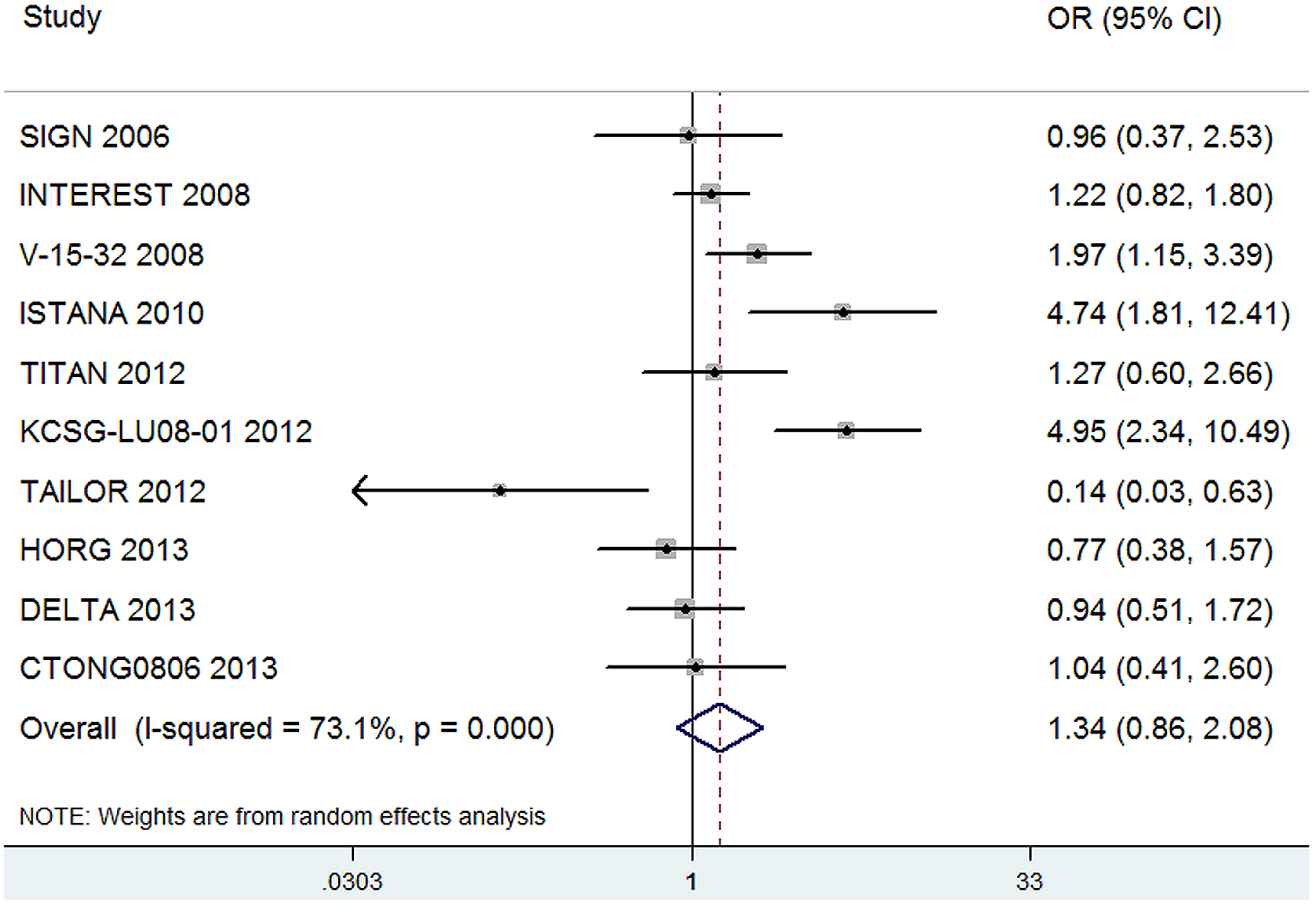
Comparison of ORR between EGFR-TKIs and chemotherapy.

Data for objective response rate (ORR) were available from all 10 trials. The pooled OR for ORR showed that there was no significant difference between an EGFR-TKI and second-line chemotherapy (OR, 1.34; 95%CI, 0.86–2.08; P = 0.20, Figure 4). Between-study heterogeneity was significant (I^2^ = 73.1%, P<0.001), and the pooled OR for ORR was performed through random effect model.

### Subgroup Analysis Based on EGFR Mutation Status

**Figure 5:**
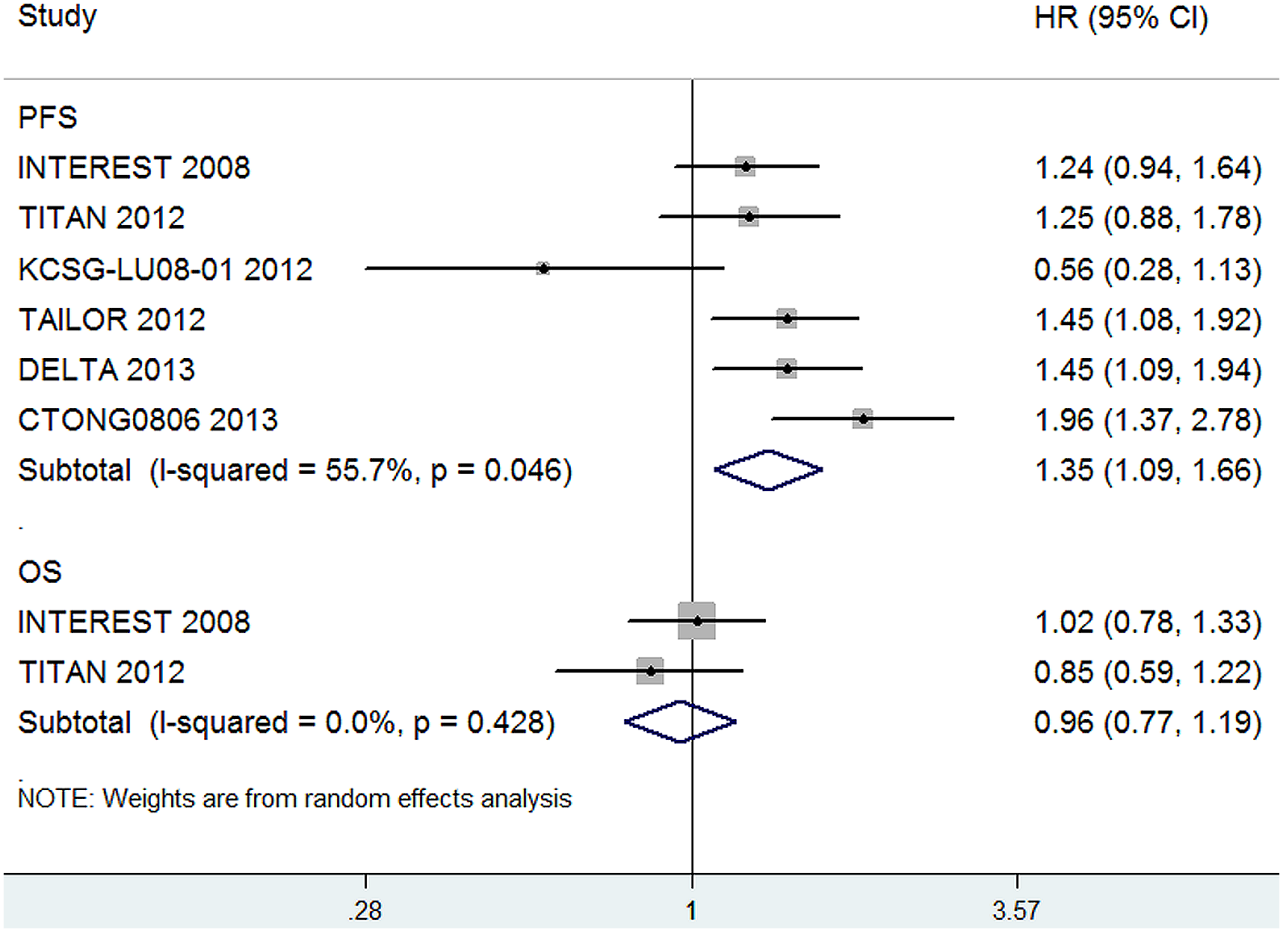
Comparison of PFS and OS between EGFR-TKIs and chemotherapy in subgroup of EGFR M^−^ patients.

Six trials [7], [11], [12], [13], [22], [25] reported HRs for PFS of EGFR M^−^ lung cancer and 2 [7], [22] reported HRs for OS of EGFR M^−^ lung cancer. Totally, the reported number of EGFR M^−^ patients was 1119. The pooled HR for PFS and OS showed that there was a significant improvement in PFS for second-line chemotherapy compared with EGFR-TKI therapy for EGFR M^−^ patients (HR, 1.35; 95%CI, 1.09–1.66; P = 0.01, Figure 5), whereas the OS between them was not significantly different (HR, 0.96; 95%CI, 0.77–1.19; P = 0.69, Figure 5).

**Figure 6:**
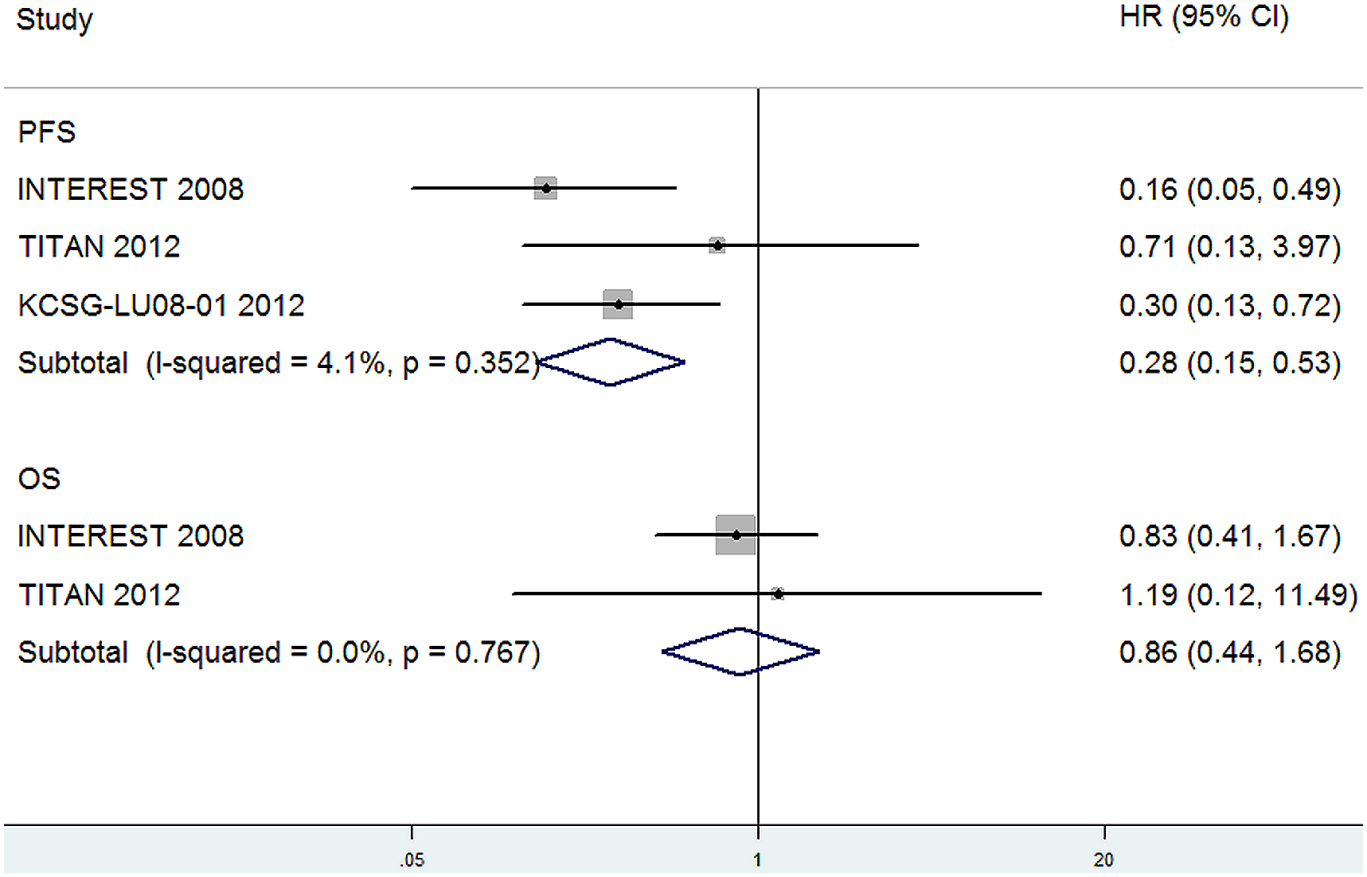
Comparison of PFS and OS between EGFR-TKIs and chemotherapy in subgroup of EGFR M^+^ patients.

Data for PFS of EGFR mutation-positive (EGFR M^+^) lung cancer were available from 3 trials [7], [22], [25] and data for OS of EGFR M^+^ lung cancer were available from 2 trials [7], [22]. Totally, the reported number of EGFR M^+^ patients was 150. The pooled HRs for PFS and OS showed that there was a significant improvement in PFS for EGFR-TKI therapy compared with second-line chemotherapy for EGFR M^+^ patients (HR, 0.28; 95%CI, 0.15–0.53; P<0.001, Figure 6), whereas the OS between them was not significantly different (HR, 0.86; 95%CI, 0.44–1.68; P = 0.65, Figure 6).

### Toxicity Analysis Results

Drug-related toxicity was described as patient-experienced grade 3–4 toxicity in this analysis. The main toxicities of these trials are listed in table 2. Compared with chemotherapy, EGFR-TKIs led to more grade 3–4 rash (OR, 7.55; 95%CI: 3.97–14.37; P<0.001). Additionally, compared with chemotherapy, a statistically significant decrease in fatigue/asthenia disorder, leukopenia and thrombocytopenia was observed (OR, 0.45; 95%CI: 0.32–0.64; P<0.001; OR, 0.04; 95%CI: 0.01–0.10; P<0.001; OR, 0.25; 95%CI: 0.08–0.83; P = 0.02, respectively). With regard to the risk of grade 3–4 diarrhea, nausea, vomiting, and anemia, equivalent frequencies were found between the EGFR-TKI arm and the chemotherapy arm. The analyses of toxicities were performed on the fixed effect model except for leukopenia (because of heterogeneity).

**Table 2:** Comparison of grade 3–4 toxicities between EGFR-TKIs and chemotherapy.

Grade 3–4 toxicity	Included trials	OR and 95%CI	P value	Heterogeneity	
				I^2^	P value
Rash	9	7.55 (3.97, 14.37)	<0.001	26.7	0.21
Diarrhea	9	1.09 (0.68, 1.74)	0.73	0.0	0.61
Fatigue/Asthenia disorder	9	0.45 (0.32, 0.64)	<0.001	4.6	0.40
Nausea	8	0.60 (0.32, 1.13)	0.12	0.0	0.68
Vomiting	8	0.79 (0.37, 1.67)	0.54	0.0	0.65
Anemia	6	0.68 (0.40, 1.14)	0.15	1.6	0.41
Leukopenia	9	0.04 (0.01, 0.10)	<0.001	74.4	<0.001
Thrombocytopenia	5	0.25 (0.08, 0.83)	0.02	0.0	0.53

### Publication Bias and Sensitivity Analysis

Egger’s test was used to check potential publication bias and the results showed that no evidence of publication bias exists (P = 0.95 for PFS, P = 0.11 for OS and P = 0.73 for ORR). The symmetry Begg’s funnel plots indicated that there was no evidence of publication bias in our meta-analysis (Figure S2). Sensitivity analysis indicated that the results of this pooled analysis were not affected by exclusion of a particular trial from the analysis.

## Discussion

Standard first-line treatment for advanced lung cancer usually consists of platinum-based doublet chemotherapy, but progression eventually occurs for most patients [27], [28]. Available second-line treatment options for patients who have failure of first-line treatment include targeted therapy or further chemotherapy. In the second-line setting, an EGFR mutation status examination was thought to be time-consuming and unnecessary. Recent studies about second-line treatment found that chemotherapy was superior to EGFR-TKIs in PFS for EGFR M^−^ NSCLC [11], [12], [13]. Our meta-analysis combined 3825 patients from 10 randomized trials. Our analysis demonstrated that regardless of EGFR mutation status, EGFR-TKIs and second-line chemotherapy had equivalent efficacy for pretreated advanced NSCLC patients. In addition, our analysis demonstrated that in terms of PFS, chemotherapy showed a significant improvement for EGFR M^−^ patients compared with EGFR-TKIs, and EGFR-TKIs were superior to chemotherapy for EGFR M^+^ patients. Overall, these data suggest that obtaining information on EGFR mutational status before initiation of second-line treatment is worth it.

Previously, the meta-analysis by Qi et al. also demonstrated both EGFR-TKIs and chemotherapy had comparable efficacy. However, the potential effect of EGFR mutation status on PFS, OS was not analysed in the analysis [9]. Subsequently, the meta-analysis by Lee et al. investigated the impact of EGFR-TKIs on PFS and OS in NSCLC and showed that an EGFR mutation is a predictive marker of PFS in all settings. However, of the 7 second-line studies included in their meta-analysis, only 5 studies compared an EGFR-TKI with chemotherapy [10]. A meta-analysis comparing EGFR-TKIs with chemotherapy as second-line therapy for wild-type EGFR lung cancer patients was presented in part at the 2013 ASCO annual meeting and also demonstrated chemotherapy had superiority in PFS over EGFR-TKIs for EGFR M^−^ patients. However, that meta-analysis only included 3 trials [14]. Our meta-analysis combined 1269 patients with explicit EGFR mutation status. Although the reported number of EGFR M^+^ patients was only 150 in these trials and caution should be used when these results are interpreted, our meta-analysis provides information to better the relation of second-line therapy and EGFR mutation status.

Our analysis suggested that PFS favored chemotherapy among pretreated EGFR M^−^ patients, whereas it favored EGFR-TKIs among those with EGFR M^+^ tumors. The possible explanation is that EGFR mutation may be a predictive biomarker for benefit of EGFR-TKIs over chemotherapy beyond first-line treatment. The IPASS trial suggested that the presence of an EGFR mutation is the strongest predictor for benefit of gefitinib in the first-line setting [29]. In IPASS, the PFS benefit of gefitinib was limited to EGFR M^+^ patients and gefitinib was associated with poorer PFS than carboplatin–paclitaxel for EGFR M^−^ patients [30]. The above combined with the results of our study suggests that the predictive value of EGFR mutation may be applied to both first-line and second-line treatment.

In our analysis, the prolonged PFS advantage in different EGFR mutational status didn’t translate into an OS advantage. This is mostly because of the high crossover rate after progression. None of these trials prohibited patients from crossing over to the other group. For example, in the INTEREST trial, of the patients in the gefitinib arm, 31% received docetaxel as subsequent therapy, and of the patients in the docetaxel arm, 37% received an EGFR-TKI subsequently [22]. Additionally, in the V-15-32 trial, 36% of patients in the gefitinib arm received subsequent docetaxel, 53% of patients in the docetaxel arm received subsequent gefitinib [23]. The high crossover rate confounded the interpretation of OS. Since there are more and more active agents emerging in the treatment of NSCLC, a PFS advantage is rarely associated with an OS advantage any more [31], [32]. Considering patients’ benefit and ethical issues, crossover treatment may be inevitable. So PFS should be deemed as a good end point. More work is still required to demonstrate the impact of PFS on OS.

Regarding grade 3–4 toxicity data, our analysis demonstrated that although EGFR-TKIs produced more rash, they produced less fatigue/asthenia disorder, leukopenia and thrombocytopenia than second-line chemotherapy. Since most of rash can be managed, as far as toxicity profiles are concerned, an EGFR-TKI is favourable. Since the toxicity profiles of EGFR-TKIs are manageable, and the combination of EGFR-TKIs with chemotherapy has shown advantage as first-line treatment [33] and as adjuvant treatment [34], this therapeutic pattern should be explored in this setting.

Several limitations should be noted from this meta-analysis. To begin with, like many other meta-analyses, this is a meta-analysis based on published data as well, so caution should be used when the results are interpreted. Secondly, the assessed EGFR M^+^ patients were only 150, which restricted our results. Thirdly, the methods for detecting EGFR mutation of these trials were not unified. For example, direct gene sequencing was used to detect EGFR mutation in most trials, while polymerase chain reaction was used in the HORG trial [26]. Different methods have different sensitivity in detecting EGFR mutations. Additionally, several trials were not 100% second-line setting studies, and geographic origin was another concern. Further prospective studies are needed to confirm the best treatment in the second-line setting for advanced NSCLC.

In conclusion, based on this meta-analysis, treatment with chemotherapy can prolong PFS in EGFR M^−^ patients, whereas has no impact on OS. EGFR-TKIs seem superior over chemotherapy as second-line therapy for EGFR M^+^ patients. It is worthwhile to obtain information on EGFR mutational status before initiation of second-line treatment. These results, combined with the toxicities, should be taken into consideration in the second-line treatment.

## Supporting Information

Figure S1 **PRISMA Flow Diagram.**
(DOC)

Figure S2 **Begg’s funnel plots of publication bias.**
(TIF)

Checklist S1 **PRISMA Checklist.**
(DOC)
