# Supplementary material for: Meta-Analysis of EGFR Tyrosine Kinase Inhibitors Compared with Chemotherapy as Second-Line Treatment in Pretreated Advanced Non-Small Cell Lung Cancer
Source: PLoS One. 2014 Jul 16;9(7):e102777. doi: 10.1371/journal.pone.0102777 (PMC4100920; doi:10.1371/journal.pone.0102777)
Supplement: Figure S1 — PRISMA Flow Diagram. (DOC) [file pone.0102777.s001.doc]

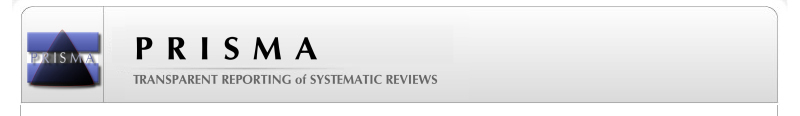
**PRISMA 2009 Flow Diagram**

**Screening**

**Included**

**Eligibility**

**Identification**

Records identified through database searching
(n = 758)

Additional records identified through other sources
(n = 619)

Records after duplicates removed
(n = 204)

Records screened
(n = 1173)

Records excluded
(n = 1072)

Full-text articles assessed for eligibility
(n =101)

Full-text articles excluded, with reasons
(n = 91)

Studies included in qualitative synthesis
(n = 10)

Studies included in quantitative synthesis (meta-analysis)
(n = 10)
