# Supplementary figures and images for: Meta-Analysis of EGFR Tyrosine Kinase Inhibitors Compared with Chemotherapy as Second-Line Treatment in Pretreated Advanced Non-Small Cell Lung Cancer
Source: PLoS One. 2014 Jul 16;9(7):e102777. doi: 10.1371/journal.pone.0102777 (PMC4100920; doi:10.1371/journal.pone.0102777)

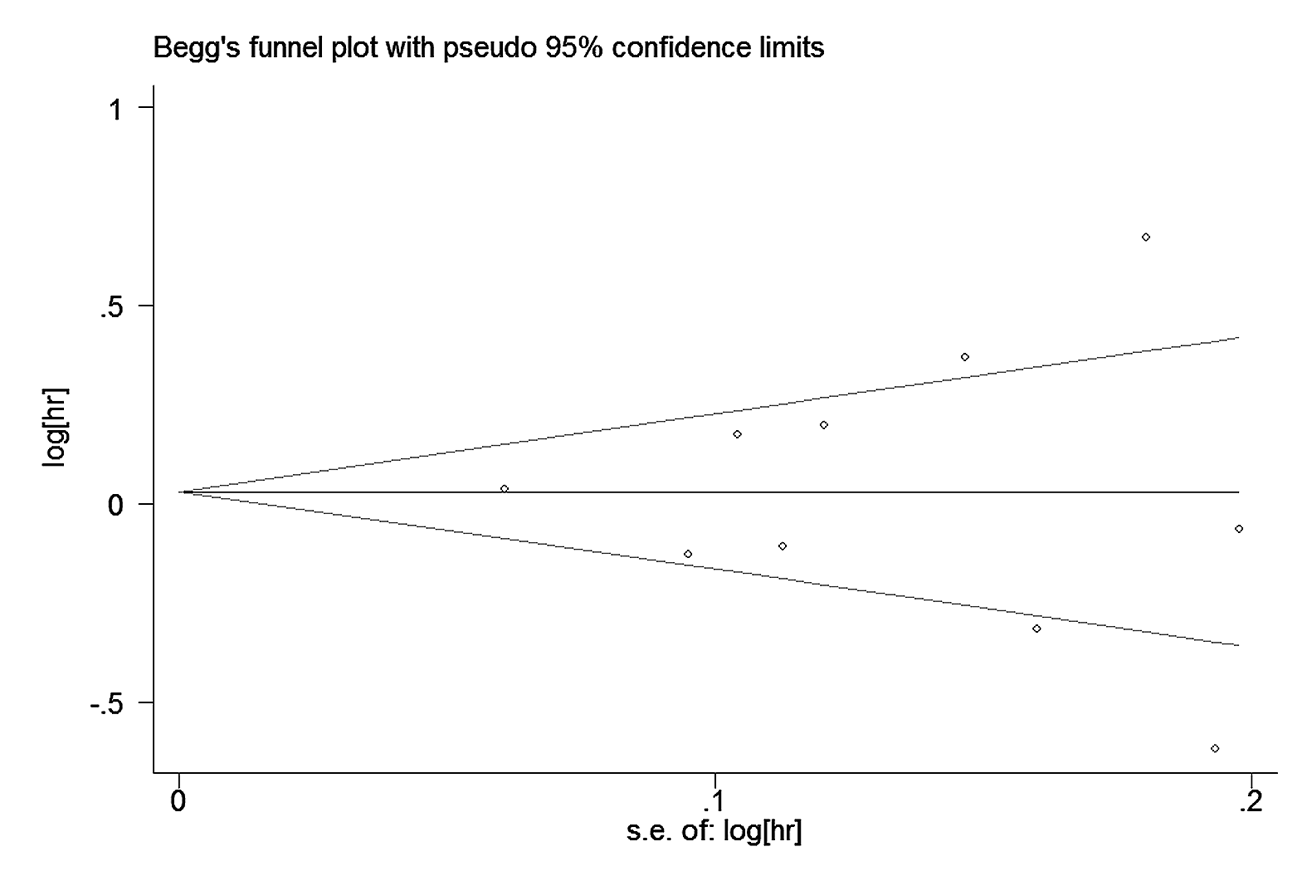

Supplement: Figure S2 — Begg’s funnel plots of publication bias. (TIF) [file pone.0102777.s002.tif]
